# Social and Demographic Factors Influencing Maternal Breast Milk Intake in Preterm and Very Low Birthweight Infants

**DOI:** 10.21203/rs.3.rs-6486705/v1

**Published:** 2025-05-08

**Authors:** Thao Ho, Amornrat Sawangkum, Alexandra Hoeman, Willow Goff, Xiaoqi Sun

**Affiliations:** University of South Florida; University of South Florida; University of South Florida; University of South Florida; Memorial Healthcare System

## Abstract

**Objective:**

Identify demographic and social factors that influence the availability of maternal breast milk (MBM) to reduce barriers and improve outcomes for very low birthweight preterm infants.

**Study Design:**

This prospective cohort study analyzed demographic, socioeconomic, and clinical data from 300 maternal-infant dyads with infants born <1500 g. Data included residential distance from the hospital, comorbidities, and infant MBM intake measured as a percentage of total enteral intake.

**Results:**

Bivariate analysis revealed that maternal race, median income by zip code, marital status, and residential distance were significantly associated with MBM intake.

In a multivariate regression model, only residential distance and marital status remained significant predictors, with greater distance from the hospital and marriage status associated with higher MBM intake.

**Conclusion:**

Residential distance from the hospital was not a significant barrier to breastfeeding in the study population.

## Introduction

The many benefits of maternal breast milk (MBM) consumption in infants are well-documented. MBM promotes the neurodevelopment of preterm infants (1, 2), a population at increased risk of neurological deficits. MBM also enhances the immunological and microbiome development in infants, reducing the risk of gastrointestinal and respiratory tract infections throughout the lifespan (3–5). Numerous studies show that MBM provides greater benefits than donor breast milk, a common feeding option for preterm infants when MBM is not available (6, 7). Mothers of preterm infants face unique challenges in initiating and sustaining breast milk supply. Infants requiring neonatal intensive care unit (NICU) admission are often unstable and cannot undergo breastfeeding and/or have diminished ability to coordinate suckling and swallowing at the breast (8). Additionally, the NICU environment can induce stress in both the infant and the mother, potentially inhibiting the initiation of breastfeeding and reducing MBM production (9). Given such challenges, it is critical to minimize other non-clinical barriers to breastfeeding in preterm and very low birthweight infants.

Numerous non-clinical maternal factors influence the continuation of MBM feedings. Recent studies suggest that infants born to mothers of lower educational attainment are breastfed for a shorter duration (10–12) Additionally, mothers with a greater household income demonstrate increased rates of exclusive breastfeeding, possibly due to improved access to lactation consultants, supplies, and education regarding the benefits of MBM (13, 14). Race and ethnicity may also play a role in MBM intake as some studies indicate that Hispanic and/or Black mothers have a higher risk of exclusive formula feeding than their White counterparts (15–17). However, other reports find that such disparities are diminished when controlling for maternal geographic residence (18).

While not robustly examined in the current literature, maternal geographic location may be a stronger predictor of MBM feedings than race and ethnicity. Therefore, establishing geographic location or distance from the hospital as a potential barrier to breastfeeding may help hospitals implement solutions for at-risk mothers, such as transportation programs or mobile lactation support. We aimed to examine the influence of geographic distance from the hospital on MBM availability in preterm infants in addition to demographic and socioeconomic factors. This information will guide clinicians and hospitals to better support mothers in providing MBM for their preterm or very low birthweight infants.

## Methods

### Study design and patient population

This was a prospective observational study of infants born at < 33 weeks of age or with birthweight < 1500 g admitted to the NICU at Tampa General Hospital between 2016 and 2022 to study nutrition and outcomes. Infants were included if the maternal residential address on file in the electronic medical record (EMR) was located within the state of Florida. Exclusion criteria were infants born to mothers with positive urinary drug screens or contraindications to breastfeeding, such as human immunodeficiency virus or human T-cell lymphotropic virus positivity or with active lesions of herpes simplex virus. Infants with classic galactosemia were also excluded from the study.

#### Data collection

MBM consumption was defined as the percentage by volume of MBM consumed enterally out of the total enteral intake. Feeding data was recorded weekly and the cumulative percentage of MBM consumption from birth until discharge was calculated as the primary dependent variable. Infant gestational age, maternal gravida, race, presence of maternal comorbidities (defined as presence of mental health diagnoses, hypertension, preeclampsia, or diabetes), and maternal marital status were also obtained. The demographic data and the maternal residential address including the 5-digit ZIP code (as defined by the United States Postal Service) were extracted from the EMR. Maternal residential distance was recorded as the distance in miles from the maternal residential address to the address of Tampa General Hospital. Distance from the hospital is used interchangeably with distance from the NICU. Additionally, yearly median household income for each ZIP code was determined from the United States Census Bureau database using the 2021 American Community Survey 5-Year Estimates (19).

### Statistical analysis

Data descriptions were expressed in mean and standard deviations (SD) or median and interquartile range (IQR) for continuous variables and in percentages for categorical variables. The associations between categorical variables were evaluated by the Fisher exact test. Independent samples *t*-tests/ANOVA and Mann Whitney U/Kruskal-Wallis test were used to compare continuous variables for normally distributed and skewed data, respectively. Statistical interactions were tested based on research questions. Regression models were tested to examine the relationship between infant MBM intake and maternal residential distance, controlling for gestational age, gravida, birthweight, maternal age, marital status, race, mode of delivery, presence of infant comorbidities, presence of maternal comorbidities, and median income by residential ZIP code. The 95% confidence intervals were used to describe the precision of the estimates. The two-tailed statistical tests were considered significant at *p* < 0.05. All analyses were performed using IBM SPSS statistical software package (IBM Corp. released 2023, IBM SPSS Statistics, Version 29.0 Armonk, NY:IBM Corp).

## Results

### Patient characteristics

Final analysis consisted of 300 infants who met the study criteria. Mean birth gestational age and birth weight were 29 ± 3 weeks and 1267 ± 427 g, respectively. The average residential distance from the hospital was 30 ± 31 miles. 55% of mothers had maternal comorbidities and 57% of infants had comorbidities during their NICU stay. The maternal race distribution was 50% White, 33% Black, and 17% other or unknown (Table 1).

### Reported maternal race

Self-reported maternal race (Black, White, and others) was strongly associated with distance from the hospital (χ(2) = 42.37, p < 0.001) and median income by zip code (χ(2) = 32.17, p < 0.001). White mothers lived further from the hospital compared to Black mothers (median 28.9 [14.5, 49.4] vs. 11.6 [7.8, 23.6], p < 0.001) and other-race mothers (14.5 [10.9, 30.7], p = 0.003). MBM intake (%) was higher in infants of White mothers compared to infants of Black mothers (85% [34%, 99%] vs. 61% [17%, 94%], p = 0.029) (Fig. 1). Black mothers had greater numbers of pregnancies (3 [1, 4] vs. 2.4 [1, 3], p = 0.001) and lower maternal ages at current pregnancy (27 [22, 32] vs. 29 [25, 34], p = 0.016) compared to White mothers. There was a greater percentage of Black mothers who were not married compared to White mothers (71% vs 37%, p < 0.001).

#### Maternal age

Maternal age was analyzed in categories of advanced maternal age (defined as ≥ 35 years) and non-advanced maternal age (defined as <35 years). Advanced maternal age was associated with a greater number of pregnancies (2 [1, 4] vs 3 [2, 5], p < 0.001), but not with infant MBM intake.

#### Marital status

About half (47%) of the mothers were married. Married mothers were older (30.7 ± 5.4 vs. 27.0 ± 5.7 years, p < 0.001), lived further from the hospital (23.1 [13, 42.5] vs. 14.2 [9.1, 35.5] miles, p = 0.001), had fewer pregnancies (2 [1, 3] vs. 3 [1, 4], p = 0.008), and had higher median income by zip code ($64K [48K, 78K] vs. $45K [40K, 57K], p < 0.001) compared to single mothers. Infants born to married mothers had significantly higher MBM intake (92% [52%, 99%] vs. 50% [18%, 89%], p < 0.001) (Fig. 2).

### Median income by residential ZIP code

Median income by residential ZIP code was correlated with MBM intake (r = 0.139, p = 0.016) and maternal age (r = 0.245, p = < 0.001) by Spearman’s correlation. Mothers from low-income households (defined as <$60,000 per year) had a greater number of pregnancies (3 [1, 4] vs 2 [1, 3], p = 0.011), younger ages at current pregnancy (27 [23, 31.5] vs. 31 [27, 34], p < 0.001), and their infants had lower MBM intake (65% [29%, 96%] vs. 86% [24%, 99%], p = 0.046) (Fig. 3).

### Residential distance from hospital

The median distance was 21 (10.5, 39.2) miles. About 50% of the families were ≤ 20 miles, 75% of families were ≤ 30 miles, and 90% of families were ≤ 60 miles from the hospital. Distance from the hospital was positively associated with infant MBM intake (Kruskal-Wallis test, χ (3) = 22.52, p < 0.001) (Fig. 4).

### Infant MBM intake

About 23% infants received ≤ 25% MBM, 38% infants received ≤ 50% MBM, and 50% infants received ≤ 75% MBM during their NICU stay. Maternal age was significantly associated with MBM intake; infants who received ≤ 25% MBM or >75% MBM were born to older mothers (Kruskal-Wallis test, χ^2^(3) = 19.75, p <0.001). Residential distance was greater (22.2 [10.9, 47.6] vs. 14.4 [10.3, 26.6] miles, p = 0.019) and birth weight was lower (1223 ± 430 vs. 1371 ± 463 g, p = 0.034) in infants who received >75% MBM compared to infants who received ≤25% MBM.

For the logistic regression model, we defined “low MBM” group consisting of infants with MBM intake ≤ 75% and “high MBM” group consisting of infants with MBM intake >75%. Clinical and demographic factors (gestational age, mode of delivery, presence of infant comorbidities, presence of maternal comorbidities, gravida, maternal age, and maternal race) and socioeconomic factors (marital status, maternal residential distance, and median income by residential ZIP code stratified as less than or greater than $60,000 USD per year) were included in the logistic regression model. The logistic regression model demonstrated that further distance from the hospital and marital status were significant predictors of MBM intake > 75% in our preterm infants (B = 0.015, 95% CI [1.005–1.024], p=0.002 and B = 1.293, 95% CI [2.001, 6.638], p < 0.001 respectively), when controlling for the above factors. The variance inflation factor for distance, income, race and marital status was 2.4 using McFadden’s R^2^.

## Discussion

The current literature indicates that race, income, marital status, and maternal age are factors that may impact MBM production, breastfeeding initiation, and duration of breastfeeding (13, 15, 16, 20). In this study, mothers living closer to the hospital more often identified as Black or unmarried. In bivariate comparisons, marriage status, greater maternal age, and higher income were protective factors, while maternal self-identification as Black was a risk factor for infant reduced MBM intake, reflective of reduced MBM production. Taking these demographic and socioeconomic factors into account, residential distance and marital status remained significant predictors of infant MBM intake.

### Breastfeeding and Distance to Hospital

Currently, the relationship between residential distance to the hospital and neonatal outcomes, especially MBM intake, is largely undetermined(21). A 2021 prospective cohort study found that shorter travel times (< 30 minutes) to the hospital is positively associated with time spent in the NICU when compared with longer travel times (> 60 minutes) (22). This may suggest that mothers who live closer to the hospital spend more time with their infants and may have a greater ability to provide MBM. However, in our studied population, the opposite was observed: further residential distance was associated with higher infant MBM intake. This outcome reveals that the relationship between residential distance and breastfeeding outcome is affected by other social, economic and geographic factors. Therefore, hospitals should evaluate their own population characteristics before investing in transportation assistance.

### Breastfeeding and Maternal Age

The relationship between maternal age and breastfeeding success is not straightforward, with older mothers providing ≤25% MBM or >75% MBM to their infants. The current literature regarding breastfeeding and maternal age has found that mothers over the age of 30 years are at an increased risk of early cessation of breastfeeding when compared to mothers aged 20 to 25 years (23, 24). Interestingly, the Centers of Disease Control’s National Immunization Survey found that mothers aged 20–29 years are 6% less likely to ever breastfeed than mothers over the age of 30 years. However, younger mothers who initiated breastfeeding did so for 5.6 months longer than the older cohort (25). Therefore, our research supports the findings that advanced age is a risk factor for early cessation of breastfeeding and MBM intake, but not initiation of breastfeeding. This relationship could explain the weaker influence of maternal age on the outcomes of our study; although advanced maternal age is associated with early MBM cessation, children born to older mothers who self-identified as White and who reported higher incomes still had higher rates of MBM intake. Thus, factors such as income and race had a more powerful effect on MBM intake than maternal age.

### Breastfeeding and Marital Status

According to our study, infants born to married mothers had a higher percentage of total MBM intake. This outcome is consistent with other studies, including a recent report which found that married mothers are two times more likely to breastfeed than their unmarried counterparts (26). This effect may be modulated by numerous variables; our data showed that married mothers were more likely to have a higher income, live further from the hospital, and self-identify as White, all of which are protective factors for MBM intake. However, when controlling for such social and demographic factors, logistic regression continued to yield a positive association between marital status and high MBM intake. This suggests that marital status independently influences MBM intake.

### Breastfeeding and Income

Our study found that income is positively correlated with MBM intake, which is consistent with the current literature. Multiple studies report that mothers of low-income households are less likely to breastfeed their children than their higher-income counterparts (25, 27). A recent study found that significantly fewer low-income mothers initiated breastfeeding and continued to breastfeed beyond six months (28). This relationship is likely due to the lack of paid maternity leave among low-income earners, as well as reduced access to lactation care due to cost barriers (13). As the current literature indicates, lack of financial accessibility may strongly influence breastfeeding behaviors. While our study results indicate that low-income mothers had greater number of pregnancies and at younger age at the current pregnancy, but income itself was not a predictor for MBM intake after controlling for other factors.

### Breastfeeding and Race

Consistent with the current literature, our study found a significant relationship between race and MBM intake. Numerous studies report that those who self-identify as Black have lower rates of breastfeeding initiation and duration compared to those who identify as White. Patel et al. theorizes that this disparity arises as Black mothers are more likely to be low-income and sole supporters of households (17). However, another study found that lower rates of MBM intake in infants of Black mothers persist despite controlling for such social and economic variables. Instead, the researchers hypothesized that this group introduces solid food earlier than other racial groups, which could explain their lower rates MBM consumption (29). This explanation supports our finding that maternal race is a powerful predictor of MBM intake which may overcome other protective factors such as geographic proximity to healthcare.

### Strengths, Weaknesses, and Future Directions

Strengths of this study include prospective longitudinal data from a diverse patient population, which allowed for analysis of various demographic characteristics. However, this study was limited by the inability to assess social support or educational status, which may play a significant role in milk production and the facilitation of breastfeeding in the NICU.

## Conclusions

Identifying barriers to breastfeeding in the NICU is important to improve health outcomes for preterm infants. Our study demonstrated that preterm infants born to mothers who resided closer to the hospital took less MBM than those with mothers who resided farther from the hospital. Marital status was also a strong predictor of MBM consumption. Mothers whose infants had less MBM milk were more likely to self-identify as Black and have a lower income status, demographic variables identified as risk factors for breastfeeding. These suggest that hospitals may need to invest in supports other than transportational solutions to increase MBM supply to preterm infants. Additional research into the relationships between marital status, social networks, and breastfeeding may provide useful information for clinicians to support mothers in breastfeeding.

## Figures and Tables

**Table 1:** Demographics and perinatal characteristics

Characteristics	N = 300
Gestational age (weeks, mean, SD)	29 ± 3
Minimum, maximum (weeks)	23, 37
Birthweight (g, mean, SD)	1273 ± 436
Minimum, maximum (g)	460, 2750
Maternal residential distance (miles, mean, SD)	30 ± 31
Minimum, maximum (miles)	2.1, 178
Maternal age (years, mean, SD)	29 ± 6
Advanced maternal age (≥35 years), *n* (%)	58 (19)
Vaginal delivery, *n* (%)	224 (75)
Cesarean delivery, *n* (%)	76 (25)
White, *n* (%)	150 (50)
Black, *n* (%)	99 (33)
Other races, *n* (%)	51 (17)
Maternal comorbidities, *n* (%)	164 (55)
Infant comorbidities, *n* (%)	172 (57)
Length of hospital stay (days, mean, SD)	61 ± 40

## Data Availability

De-identified data will be available upon request.
